# Effect of Mn Addition on the Microstructures and Mechanical Properties of CoCrFeNiPd High Entropy Alloy

**DOI:** 10.3390/e21030288

**Published:** 2019-03-16

**Authors:** Yiming Tan, Jinshan Li, Jun Wang, Hongchao Kou

**Affiliations:** State Key Laboratory of Solidification Processing, Northwestern Polytechnical University, Xi’an 710072, China

**Keywords:** high entropy alloys, solidification, alloy design, eutectic dendrites, hierarchical nanotwins

## Abstract

CoCrFeNiPdMn*_x_* (*x* = 0, 0.2, 0.4, 0.6, 0.8) high entropy alloys (HEAs) were prepared and characterized. With an increase in Mn addition, the microstructures changed from dendrites (CoCrFeNiPd with a single face-centered-cubic (FCC) phase) to divorced eutectics (CoCrFeNiPdMn_0.2_ and CoCrFeNiPdMn_0.4_), to hypoeutectic microstructures (CoCrFeNiPdMn_0.6_), and finally to seaweed eutectic dendrites (CoCrFeNiPdMn_0.8_). The addition of Mn might change the interface energy anisotropy of both the FCC/liquid and MnPd-rich intermetallic compound/liquid interfaces, thus forming the seaweed eutectic dendrites. The hardness of the FCC phase was found to be highly related to the solute strengthening effect, the formation of nanotwins and the transition from CoCrFeNiPd-rich to CoCrFeNi-rich FCC phase. Hierarchical nanotwins were found in the MnPd-rich intermetallic compound and a decrease in either the spacing of primary twins or secondary twins led to an increase in hardness. The designing rules of EHEAs were discussed and the pseudo binary method was revised accordingly.

## 1. Introduction

High entropy alloys (HEAs) [1] or multi-principal element alloys [2] are now attracting more and more attention [3,4,5,6,7,8,9,10]. In contrast to the traditional alloys with one principal element or two, HEAs have at least four principal elements and usher in an expansive alloy space for exploring potential new materials with brilliant properties [11,12,13,14,15,16,17,18,19,20,21,22,23,24,25,26]. Initially, studies of HEAs concentrated to a greater extent on the solid-solution phases, e.g., the HEAs with a single face-centered-cubic (FCC) phase, with a single body-centered-cubic (BCC) phase or with dual FCC and BCC phases. Lots of studies suggested that the high configurational entropy would be able to stabilize thermodynamically the solid-solution phases [1,4,5,27,28]. As the researches move forward, more and more studies suggested that the high configurational entropy alone could not determine completely the constituent phases, because most of the HEAs consisted of multi-phases [29,30,31,32,33,34].

Although the HEAs with a single solid-solution phase have some advantages (e.g., higher melting points than the HEAs with multi-phases, higher strength for the HEAs with a single BCC phase, better ductility for the HEAs with a single FCC phase etc.), their good properties are usually accompanied by some disadvantages, which are fatal for technological applications. One is that the HEAs with a single solid-solution phase usually have inadequate liquidity, poor castability and hence considerable chemical inhomogeneity [21,35]. The other is that the HEAs with a single solid-solution phase could not achieve a balance between high strength and good ductility (e.g., the HEAs with a single FCC phase were ductile but not strong enough while the HEAs with a single BCC phase were adequately strong but at risk of brittleness [21,35,36,37]).

To tackle the aforementioned problems, eutectic HEAs (EHEAs) [21] were proposed. On the one hand, EHEAs should have the general character of traditional eutectic alloys. In this sense, EHEAs should have better fluidity and thus better castability and less casting defects [15,35]. On the other hand, EHEAs as one kind of in-situ composites with lamellar or rod-like eutectic microstructures might reach the balance between strength and ductility via mixing the soft FCC phase with the hard BCC phase or intermetallic compound [15,35,38,39,40,41,42,43,44]. Some EHEAs indeed have outstanding properties. Lu et al. [15] reported the AlCoCrFeNi_2.1_ EHEA with simultaneous high strength (944 MPa) and good ductility (25.6%). The excellent mechanical properties do not depend significantly on derivation of eutectic compositions [35]. After cold-rolling and annealing, its strength reached up to 1.2 GPa and its elongation could remain at about 12% [40]. After cryo-rolling and annealing, its strength could reach up to 1.47 GPa while its ductility could even increase to 14% [41]. He et al. [42,43] designed the CoCrFeNiNb*_x_* EHEAs and found that the microstructures were stable from 600 °C to 900 °C.

The current work aims to report a new EHEA. From Ref. [45], CoCrFeNiPd is a single FCC solid solution HEA. From the Mn-Pd phase-diagram [46], Mn_x_Pd_y_ is a relative stable intermetallic compound. We hence chose CoCrFeNiPd as a FCC solid solution phase and Mn_x_Pd_y_ as an intermetallic compound (IMC) phase to design pseudo binary EHEAs via adjusting the content of IMC forming element Mn to finally get the eutectic structure. The effect of Mn addition on the microstructures was investigated and a seaweed eutectic dendrite solidification microstructure was found in the CoCrFeNiPdMn_0.8_ EHEA. The effect of Mn addition on the mechanical properties was studied by nano-indentation and compression tests. The size effects of primary and secondary twins on the hardness of Mn*_x_*Pd*_y_* phase were shown. The designing rules of EHEAs were improved.

## 2. Materials and Methods 

### 2.1. Material Preparation

The ingots were prepared by arc melting under a Ti-gettered, high-purity argon atmosphere. Elements of Co, Cr, Fe, Ni, Mn and Pd with purities better than 99.95 wt.% were chosen as the raw materials. To prevent the mass loss due to evaporation of Mn, a high purity Fe-68.7at.%Mn intermediate alloy was prepared in advance and the total mass loss of each ingot was less than 0.3 wt.%. In order to ensure the chemical homogeneity, electromagnetic stirring was used during the melting process; each ingot was re-melted at least five times in the water-chilled copper crucible, held at a liquid state for at least 5 min and flipped before each melting process. The prepared button-shaped ingots were approximately 20 mm in diameter and 10 mm in thickness.

### 2.2. Material Characterization

The crystal structures were analyzed by X-ray diffraction (XRD, DX2700, Fang Yuan Company, Dandong, China) using Co k*α* radiation and a 2θ scattering range of 20°–120°. The microstructures were characterized by the field emission scanning electron microscopy (SEM, Zeiss SUPRA 55, Zeiss Inc., Jena, Germany) operated at 15 kV. The SEM samples were first polished and then etched for a few seconds within the solution of hydrochloric acid, sulfuric acid and supersaturated copper sulfuric (30 mL, 10 mL, 1 g). After the SEM observations, the samples for transmission electron microscopy (TEM) analysis were cut from the center of the SEM samples, prepared by mechanically polishing to a thickness of 45 μm, punched into disks with a diameter of 3 mm and then thinned by ion milling (GATAN 691, Gatan Inc., Warrendale, PA, United State). The chemical components and element distributions in different phases were measured by an electron probe micro-analyzer (EPMA, Shimadzu 1720, Shimadzu Inc., Kyoto, Japan) and an energy dispersive spectrometer (EDS) attached to TEM (TecnaiFG^2^).

The hardness and elastic modulus of constituent phases in the as-cast alloys were investigated by the Nano-indenter XP^®^ system (MTS Inc., Eden Prairie, MN, United State) at room temperature with a diamond Berkovich indenter at a peak load of 20 mN and a load rate of 0.1 mN·s^−1^. The peak load was held for about 5 s to eliminate the instrument noise and five different points were measured for each phase. The samples for nano-indentation were mechanically polished to 1 mm thickness and then electro-polished in an electrolyte of 90 vol.% ethanol and 10 vol.% perchloric acid, with a voltage of 30 V and a polishing time of about 20 s in Struers LectroPol-5. The compression tests were conducted at room temperature in an electronic testing machine (INSTRON 3382, Instron Inc., Norwood, MA, United State) with a strain rate of 1 × 10^−3^ s^−1^. Cuboid specimens were produced by electric-discharged machining from the cast buttons. The samples were 6 mm in height and 3 mm in length and width, giving an aspect ratio of 2. In order to show the solidification path, the thermal histories of as-cast alloys were measured by a differential scanning calorimetry (DSC, Netzsch 449 C, Netzsch Inc., Selb, Germany) under a flow of purified argon for protection and with a rate of 20 K min^−1^. The mass of samples was about 15 mg.

## 3. Results

### 3.1. Crystal Structures and Microstructures

**Figure 1:**
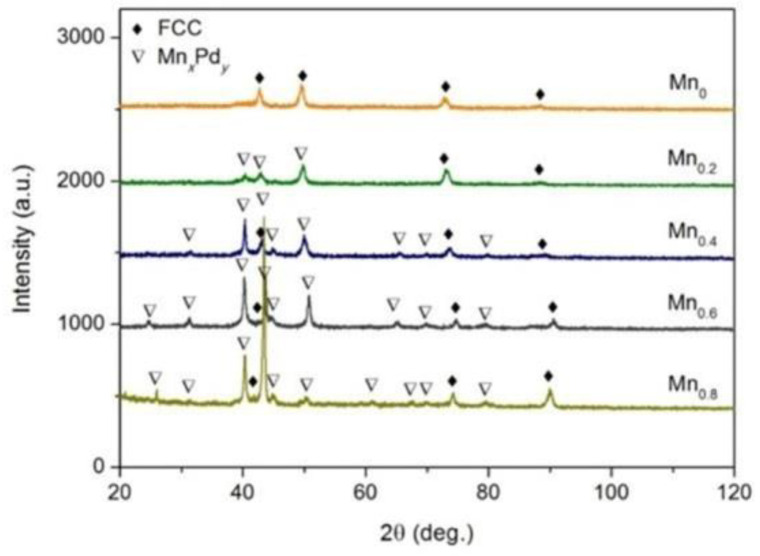
XRD patterns of as-cast CoCrFeNiPdMnx (*x* = 0, 0.2, 0.4, 0.6, 0.8) HEAs.

Figure 1 shows the XRD patterns of as-cast CoCrFeNiPdMn*_x_* (*x* = 0–0.8) HEAs. It should be noted that the CoCrFeNiPdMn*_x_* HEA in what follows was denoted as Mn*_x_* for short (e.g., Mn_0.2_ stands for the CoCrFeNiPdMn_0.2_ alloy). The Mn_0_ HEA was of a single FCC phase with a lattice parameter of *a* = 3.669 Å. The Mn_0.2_, Mn_0.4_, Mn_0.6_ and Mn_0.8_ HEAs had a dual FCC phase and Mn*_x_*Pd*_y_* intermetallic compound. Because the diffraction peaks of Mn*_x_*Pd*_y_* intermetallic compound are intensified with increasing Mn addition, one could draw a conclusion that the Mn addition promotes the formation of Mn*_x_*Pd*_y_* intermetallic compound. However, the diffraction peaks of MnPd, Mn_2_Pd_3_ and Mn_3_Pd_5_ as well as those of Mn_7_Pd_9_ and Mn_11_Pd_21_ were quite similar. The XRD results alone were therefore not able to distinguish the crystal structure of the Mn*_x_*Pd*_y_* intermetallic compound.

**Figure 2:**
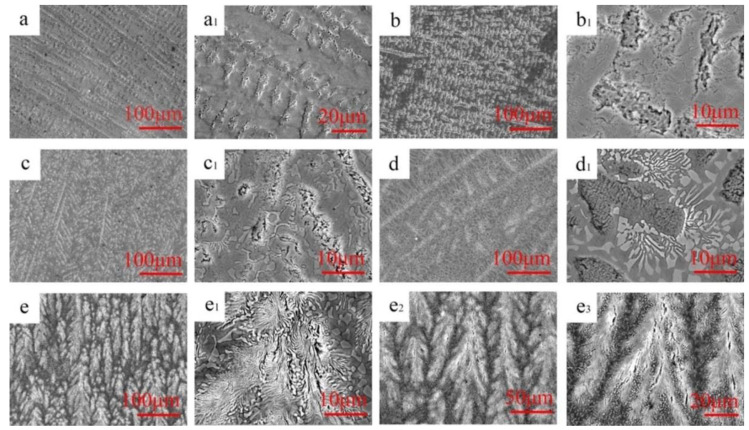
SEM images of as-cast CoCrFeNiPdMn*_x_* (*x* = 0, 0.2, 0.4, 0.6, 0.8): Mn_0_ (**a**,**a_1_**), Mn_0.2_ (**b**,**b_1_**), Mn_0.4_ (**c**,**c_1_**), Mn_0.6_ (**d**,**d_1_**) and Mn_0.8_ (**e**,**e_1_**,**e_2_**,**e_3_**).

Typical microstructures of as-cast Mn*_x_* (*x* = 0.2–0.8) HEAs are shown in Figure 2. The Mn_0_ HEA exhibited a single solid-solution phase and the coarse dendrites were of several hundred or even a thousand microns; see Figure 2a,a_1_ in different magnifications. For the Mn_0.2_ EHEA, the microstructure consisted of a main FCC solid-solution phase in the dendrite and a sporadic distributed granular Mn*_x_*Pd*_y_* intermetallic compound; see Figure 2b. Because the Mn*_x_*Pd*_y_* intermetallic compound distributed within the inter-dendrites, it could be reasonable to conclude that the microstructure belonged to divorced eutectics; see Figure 2b_1_ in which the FCC phase and the Mn*_x_*Pd*_y_* intermetallic compound are in dark grey and light grey, respectively. It should be pointed out that at the inter-dendrites, a eutectic microstructure could be found but its volume fraction was very small. The microstructure of Mn_0.4_ EHEA was quite similar to the Mn_0.2_ EHEA, except that both the volume fractions of eutectics and granular Mn*_x_*Pd*_y_* intermetallic compound were much larger; see Figure 2c,c_1_ in different magnifications. The microstructure changes from a hypoeutectic microstructure for the Mn_0.6_ EHEA (e.g., a primary FCC dendrite around which were the lamellar eutectics) to a fully eutectic microstructure for the Mn_0.8_ EHEA (e.g., a eutectic dendrite with a fine lamellar spacing around which were the coarse granular eutectics); see Figure 2d–e_1_ in different magnifications. In order to show the characteristics of the eutectic dendrite pattern in the Mn_0.8_ EHEA, two additional figures with different amplifications are shown in Figure 2e_2_,e_3_. Figure 2e_2_ shows an overall view of eutectic dendrites and Figure 2e_3_ presents some details for tip splitting of eutectic dendrites. Because the tips repeatedly split into several parts and grew on themselves, the microstructure of Mn_0.8_ HEA belonged to seaweed eutectic dendrites [47,48].

### 3.2. Phase Identification

**Figure 3:**
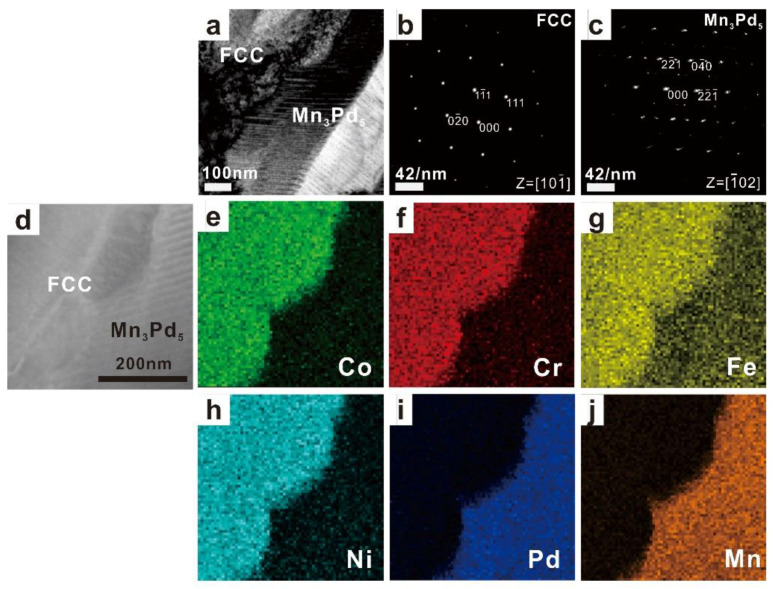
TEM images (**a**) and (**d**), the corresponding SAED patterns of FCC (**b**) and Mn_3_Pd_5_ (**c**) phases, and the EDS mapping of Co (**e**), Cr (**f**), Fe (**g**), Ni (**h**), Pd (**i**), Mn (**j**) for the as-cast CoCrFeNiPdMn_0.2_ HEA.

**Figure 4:**
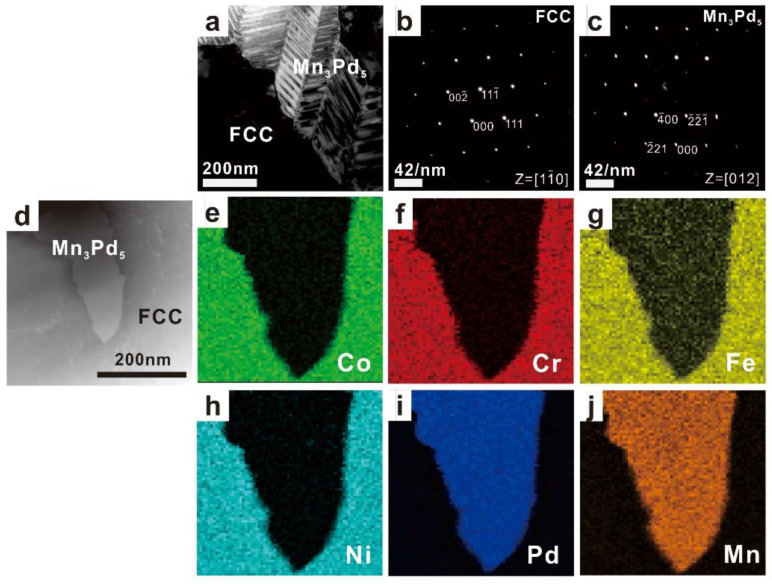
TEM images (**a**) and (**d**), the corresponding SAED patterns of FCC (**b**) and Mn_3_Pd_5_ (**c**) phases, and the EDS mapping of Co (**e**), Cr (**f**), Fe (**g**), Ni (**h**), Pd (**i**), Mn (**j**) for the as-cast CoCrFeNiPdMn_0.4_ HEA.

**Figure 5:**
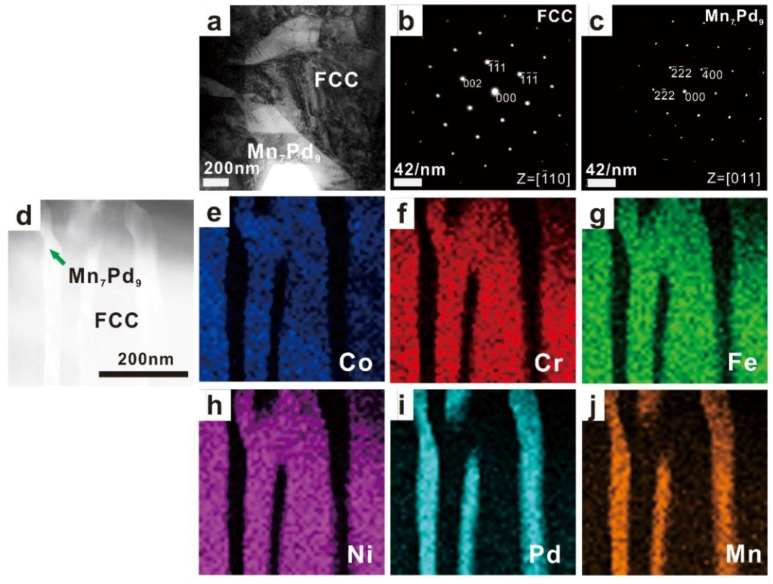
TEM images (**a**) and (**d**), the corresponding SAED patterns of FCC (**b**) and Mn_7_Pd_9_ (**c**) phases, and the EDS mapping of Co (**e**), Cr (**f**), Fe (**g**), Ni (**h**), Pd (**i**), Mn (**j**) for the as-cast CoCrFeNiPdMn_0.6_ EHEA.

**Figure 6:**
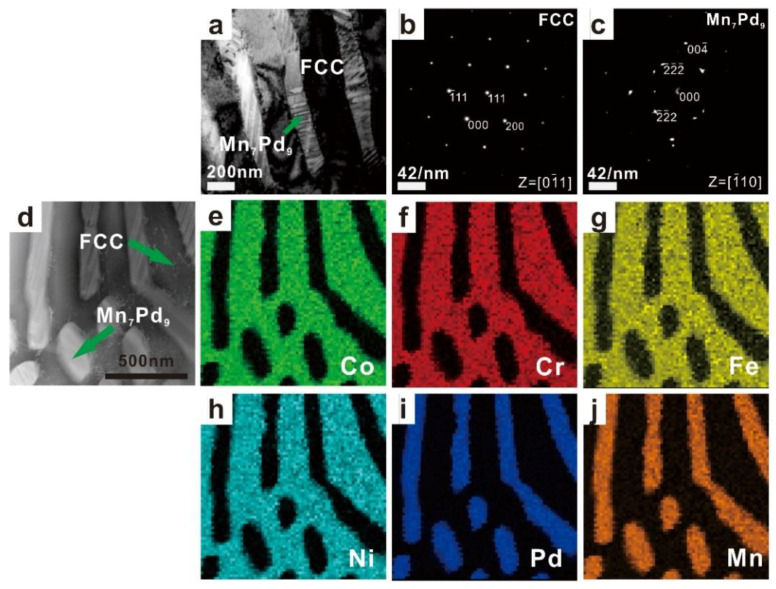
TEM images (**a**) and (**d**), the corresponding SAED patterns of FCC (**b**) and Mn_7_Pd_9_ (**c**) phases, and the EDS mapping of Co (**e**), Cr (**f**), Fe (**g**), Ni (**h**), Pd (**i**), Mn (**j**) for the as-cast CoCrFeNiPdMn_0.8_ RHEA.

The TEM results of as-cast Mn*_x_* (*x* = 0.2, 0.4, 0.6, 0.8) HEAs are shown in Figure 3, Figure 4, Figure 5 and Figure 6. In each figure, the bright-field TEM images (a, d), the selected area electron diffraction (SAED) pattern of FCC (c) and Mn*_x_*Pd*_y_* (d) phases, the EDS mapping of Co (e), Cr (f), Fe (g), Ni (h), Pd (i) and Mn (j) elements are shown. To show the effect of Mn addition on the phase transition in the Mn*_x_* HEAs, the chemical compositions of FCC and Mn*_x_*Pd*_y_* phases were measured by EDS attached to TEM and EMPA. In the current work, four points were randomly selected for each phase in the fine lamellar region by EDS and five points were measured randomly for each phase in the surrounding coarse granular eutectic region by EPMA. Because the average compositions measured by EDS and EPMA were quite close, only the EPMA results for the FCC solid-solution phase and Mn*_x_*Pd*_y_* intermetallic compound are summarized in Table 1 and Table 2, respectively.

For the Mn_0.2_ EHEA, the FCC phase was rich in Co, Cr, Fe, Ni and Pd but depleted of Mn, whereas for the Mn*_x_*Pd*_y_* phase, the compositions of Co, Cr, Fe and Ni were negligible; see Figure 3 and Table 1. According to the SAED patterns taken from the FCC-region and Mn*_x_*Pd*_y_*-region, the matrix was of a FCC structure while the Mn*_x_*Pd*_y_* phase was a Mn_3_Pd_5_ intermetallic compound with lattice parameters of *a* = 0.2285 nm, *b* = 0.1998 nm and *c* = 0.2278 nm, being consistent with the XRD results in Figure 1. It should be pointed out that even though the composition of Pd in the FCC phase (≈13.5%) was much larger than that of Mn (≈2%), it was still considerably smaller than that in Mn_3_Pd_5_ intermetallic compound (≈47%). Therefore, the fact that the FCC phase was rich in Pd cannot be shown by the EDS mapping; see Figure 3i. For the Mn_0.4_ EHEA, the same result could be found from the SAED patterns, i.e., the matrix was the FCC phase and the Mn*_x_*Pd*_y_* phase was the Mn_3_Pd_5_ intermetallic compound. The FCC phase was still a (CoCrFeNiPd)-rich one and similar EDS mappings could be found; see Figure 4e–j.

With the further addition of Mn element, the FCC phases became rich in Co, Cr, Fe and Ni for the Mn_0.6_ and Mn_0.8_ EHEAs, while the compositions of Mn (~42.3% and 41.7%) and Pd (~43.7% and ~40.4%) were comparable in the Mn*_x_*Pd*_y_* phases; see Figure 5e–j and Figure 6e–j, Table 1 and Table 2. According to the SAED patterns in Figure 5c and Figure 6c, the Mn*_x_*Pd*_y_* phase could be the Mn_7_Pd_9_ or the Mn_11_Pd_21_ intermetallic compound with lattice parameters of *a* = *b* = 0.2267 nm, *c* = 0.203 nm or *a* = *b* = 0.2235 nm, *c* = 0.1816 nm. Because the Mn_11_Pd_21_ phase was neither confirmed experimentally nor theoretically [46], the Mn*_x_*Pd*_y_* phase in the Mn_0.6_ and Mn_0.8_ EHEAs was ultimately determined to be the Mn_7_Pd_9_ intermetallic compound.

### 3.3. Solidification Path

**Figure 7:**
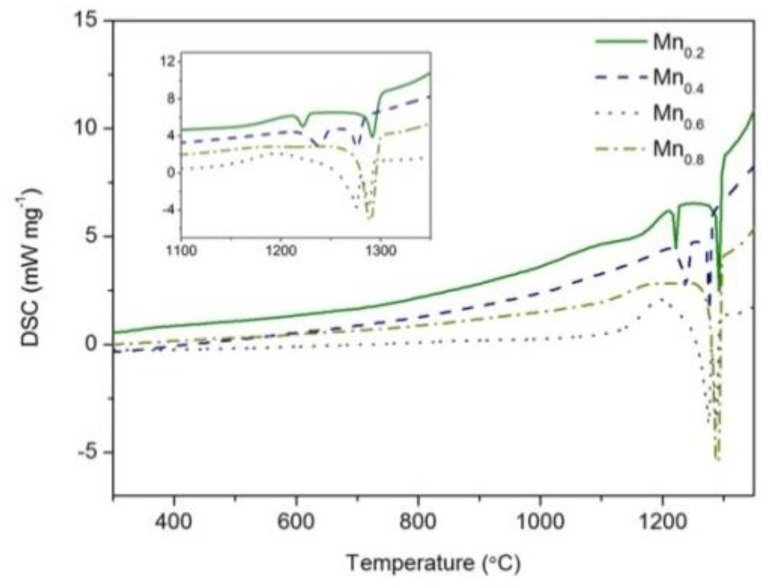
DSC solidification curves of as-cast CoCrFeNiPdMn*_x_* (*x* = 0.2–0.8) HEAs. Insert shows the magnified exothermic peaks during solidification.

To confirm further the effect of Mn addition on solidification microstructures, the cooling histories were measured by DSC; see Figure 7. For the Mn_0.2_ (the solid line) and Mn_0.4_ (the dashed line) EHEAs, two completely separated exothermal peaks could be found during the solidification process. The first and the second peak should correspond to the primary solidification of the FCC phase and following growth of the Mn_3_Pd_5_ intermetallic compound or eutectic growth. For the Mn_0.6_ EHEA (the dotted line), two exothermal peaks still exited during the solidification process but they overlapped with each other. As shown in Figure 1 and Figure 2, an increase of the Mn content promoted the formation of Mn*_x_*Pd*_y_* phase, thus intensifying the second exothermal peak during solidification and narrowed the distance between the two peaks as shown in Figure 7. For the Mn_0.8_ EHEA, only one solidification peak could be found; see the dashed-dotted line in Figure 7. The peak should correspond to eutectic solidification.

### 3.4. Mechanical Properties

**Figure 8:**
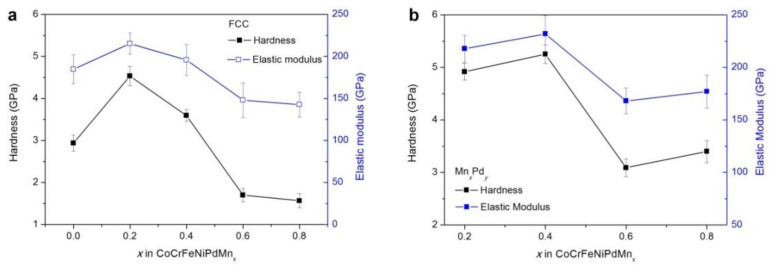
Hardness and elastic modulus of FCC phase (**a**) Mn_x_Pd_y_ phase (**b**) in the CoCrFeNiPdMn*_x_* (*x* = 0.2–0.8) HEAs.

The nanoindentor was used to measure the hardness and elastic modulus of FCC and Mn*_x_*Pd*_y_* phases; see Figure 8a,b. It should be noted that the lamellar spacing of lamellar eutectics in the Mn_0.6_ and Mn_0.8_ EHEAs was so fine that it was beyond the measurability of the nanoindentor. In this case, the coarse granular eutectics were measured. From Figure 8a, it can be seen that with an increase in the Mn addition, the hardness and elastic modulus of FCC phase first increased and then decreased. From Table 1, the FCC phase was CoCrFeNiPd-rich for the Mn_0.2_ and Mn_0.4_ EHEAs while it was CoCrFeNi-rich for the Mn_0.6_ and Mn_0.8_ EHEAs. From Ref. [49], the measured hardness of CoCrFeNiPd HEA 3.16 GPa was nearly twice of CoCrFeNi HEA 1.47 GPa. This was the reason why the hardness of Mn_0_, Mn_0.2_ and Mn_0.4_ HEAs was much larger than that of Mn_0.6_ and Mn_0.8_ HEAs; see Figure 8a. For the Mn*_x_*Pd*_y_* phase, the hardness of the Mn_3_Pd_5_ intermetallic compound in the Mn_0.2_ (4.9 GPa) and Mn_0.4_ (5.3 GPa) EHEAs was much larger than that of the Mn_7_Pd_9_ intermetallic compound in the Mn_0.6_ (3.1 GPa) and Mn_0.8_ (3.4 GPa) EHEAs. For both the FCC and Mn*_x_*Pd*_y_* phases, the evolution tendencies of hardness were the same as those of the elastic modulus.

**Figure 9:**
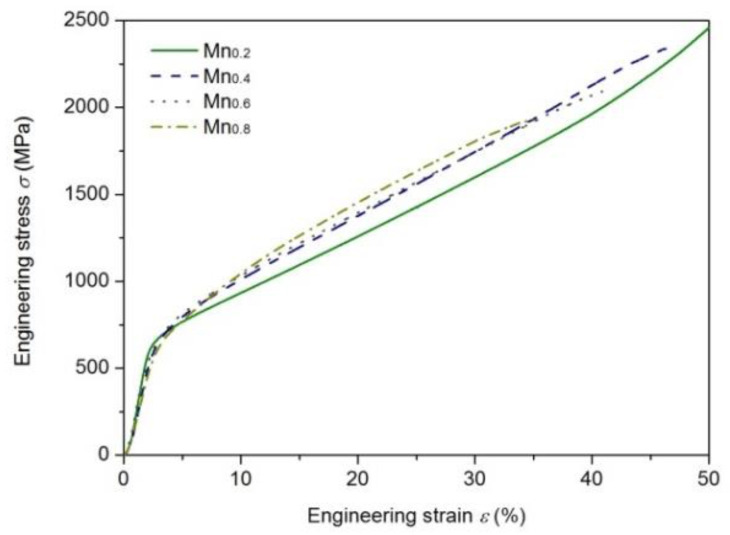
Compressive engineering stress-strain curves of as-cast CoCrFeNiPdMn*_x_* (*x* = 0.2–0.8) HEAs.

To show further the effect of Mn addition on the mechanical properties, compression tests were conducted for the as-cast Mn*_x_* HEAs; see Figure 9 One can see that with the increase of Mn addition, the yielding strength held constantly at about 650 MPa. The fracture strain (strength) decreased from about 50% (2.4 GPa) for the Mn_0.2_ HEA to about 35% (1.9 GPa) for the Mn_0.8_ HEA. The current EHEAs had good strength and ductility.

## 4. Discussion

### 4.1. Effect of Mn Addition on Microstructures

With an increase in the Mn content, the microstructures of Mn*_x_* HEAs changed from dendrites for the Mn_0_ HEA to divorced eutectics for the Mn_0.2_ and Mn_0.4_ EHEAs, to hypoeutectic microstructures for the Mn_0.6_ EHEA and finally to eutectic dendrites for the Mn_0.8_ EHEA. The eutectic dendrite solidification pattern in the Mn_0.8_ EHEA was formed by cooperative growth of the FCC phase and Mn_7_Pd_9_ intermetallic compound. From Table 1 and Table 2, the FCC phase was lacking Mn while the Mn_7_Pd_9_ intermetallic compound was lacking Co, Cr, Fe and Ni. Therefore, lateral solute diffusion of Co, Cr, Fe, Ni and Mn formed the eutectic pattern while longitudinal solute diffusion of Pd made the eutectic interface unstable to a eutectic dendrite.

From Figure 2e_2_,e_3_, seaweed eutectic dendrites were found for the Mn_0.8_ EHEA. Unlike the normal dendrite pattern where the structure branches with pronounced orientation order, the seaweed pattern is characterized by tip-splitting and the key factor for its formation is weak interface energy anisotropy [50]. Generally, the formation of seaweed dendrites is highly related to alloy compositions and solidification conditions [51,52,53,54]. For example, the effect of Zn content on the microstructures of directional solidification of Al-Zn alloys was studied by X-ray tomographic microscopy and phase-field simulation [51]. Accordingly, an increase in the Zn content modified the interface energy anisotropy, thus leading to the transition from <100> dendrites at low Zn content to <110> dendrites at high Zn content, between which were the <320> seaweed dendrites. For both the undercooled Cu-8.9 wt.% Ni and Cu-3.98 wt.% Ni alloys [53,54], a transition from <100> dendrites to mixed <100> and <111> seaweed dendrites and then to <111> dendrites was reported.

For eutectic solidification that consisted of at least two solid phases, its morphology was determined by a combination effect of eutectic phases and the formation mechanism became more complex. Eutectic seaweed dendrites were reported in the undercooled Co-24.0at.%Sn eutectic alloy, in which the weak interface energy anisotropy ascribed to an alternate arrangement of lamellae and alloy physical properties [55]. For the current Mn*_x_* HEAs, primary FCC dendrites were found in the divorced eutectics (e.g., Mn_0.2_ and Mn_0.4_) and the hypoeutectic microstructures (e.g., Mn_0.6_), indicated that its interface energy anisotropy was not weak. From Table 1 and Table 2, the addition of Mn changed not only the compositions of FCC phase but also those of the Mn_7_Pd_9_ intermetallic compound. Therefore, it was quite possible that the addition of Mn influenced the interface energy anisotropy of both the FCC/liquid and Mn*_x_*Pd*_y_*/liquid interfaces, thus forming the seaweed eutectic dendrites in the Mn_0.8_ EHEA.

### 4.2. Effect of Mn Addition on Mechanical Properties

**Figure 10:**
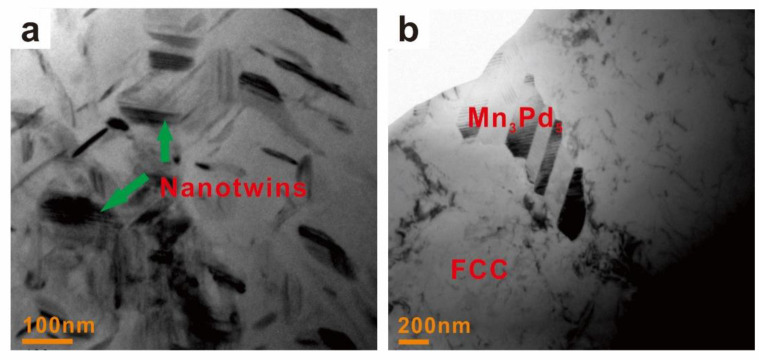
TEM images of FCC phase in the Mn_0.2_ (**a**) and Mn_0.4_ (**b**) EHEAs.

Because an increase in Mn addition results in a transition from the CoCrFeNiPd-rich to the CoCrFeNi-rich FCC phase in the Mn*_x_* HEAs (Table 1) and the hardness of CoCrFeNiPd HEA is much higher than that of CoCrFeNi HEA [49], the hardness of the FCC phase should decrease with increasing Mn addition. This was however, not the case, e.g., the hardness increased first and then decreased; see Figure 8a. The larger hardness of the FCC phase in the Mn_0.2_ and Mn_0.4_ EHEAs than that in the Mn_0_ HEA could be ascribed to the solute strengthening effect. But this effect alone cannot explain the fact that the hardness of the FCC phase in the Mn_0.2_ EHEA was larger than that in the Mn_0.4_ EHEA. The TEM results showed that a small amount of Mn addition might promote but a large amount would suppress the formation of nanotwins in the FCC phase; see Figure 10. Abundant nanotwins of about 50 nm could be found in the Mn_0.2_ EHEA, whereas for the Mn_0.4_ EHEA, it was almost free of nanotwins and so were the Mn_0.6_ and Mn_0.8_ EHEAs (not shown here). Therefore, the solute strengthening effect and the formation of nanotwins made the hardness increase first with increased Mn addition, the suppression of nanotwins then decreased the hardness and finally the transition from the CoCrFeNiPd-rich to the CoCrFeNi-rich FCC phase made the hardness decrease considerably.

Besides, hierarchical nanotwins were found in the Mn*_x_*Pd*_y_* intermetallic compounds of Mn_0.2_-Mn_0.8_ EHEAs; see Figure 10. With the help of Image-Pro Plus software, the spacing of the primary twins (*λ*_1_) and secondary twins (*λ*_2_) in the Mn*_x_*Pd*_y_* intermetallic compound were measured for the Mn_0.2_-Mn_0.8_ EHEAs; see Table 3. With an increase in the Mn addition, *λ*_1_ decreased but *λ*_2_ remained unchanged for the Mn_3_Pd_5_ intermetallic compound. For the Mn_7_Pd_9_ intermetallic compound, *λ*_1_ did not change significantly but *λ*_2_ decreased. The measured spacing of primary twins (242.1 nm, 180.3 nm) and secondary twins (10.0 nm, 9.98 nm) in the Mn_3_Pd_5_ intermetallic compound were much larger than those in the Mn_7_Pd_9_ intermetallic compound (15.0 nm, 15.0 nm for *λ*_1_, 2.22 nm and 1.46 nm for *λ*_2_) but the hardness of the former was much larger than that of the latter. However, for the same phase, a decrease of either *λ*_1_ or *λ*_2_ would increase the hardness of the intermetallic compound, being consistent with Yuan and Wu [56] who studied the size effects of primary and secondary twins on the atomistic deformation mechanisms in the hierarchically nanotwinned metals.

### 4.3. Designing Rules for EHEAs

Even though the EHEAs have good processing and mechanical properties, most of the reported EHEAs were found by the trial and error method. Up to now, several studies were carried out for designing EHEAs [42,57,58,59]. Lu et al. [57] started from their representative AlCoCrFeNi_2.1_ EHEA. They divided the constituent elements into two different groups, i.e., Al and Ni with very high negative mixing of enthalpy (−22 kJ·mol^−1^), and Co, Cr and Fe with similar atomic size and very small negative mixing of enthalpy; see Table 4. Their method was to substitute Al by Zr, Nb, Hf and Ta that had very high negative mixing of enthalpy with Ni. After using the enthalpy mixing of equimolar binary alloys to obtain the eutectic points, four new EHEAs, i.e., Zr_0.6_CoCrFeNi_2.1_, Nb_0.74_CoCrFeNi_2.1_, Hf_0.55_CoCrFeNi_2.1_ and Ta_0.65_CoCrFeNi_2.1_, were reported. In their subsequent work [58], the eutectic composition containing (Ni, Co, Cr, Fe)-rich solid-solution phase in the (Co, Cr, Fe, Ni)-(Nb, Ta, Zr, Hf) binary systems were averaged to obtain the eutectic compositions of pseudo binary alloy CoCrFeNiM*_x_* (M = Nb, Ta, Zr and Hf). Consequently, four new EHEAs, i.e., Zr_0.51_CoCrFeNi, Nb_0.6_CoCrFeNi, Hf_0.49_CoCrFeNi and Ta_0.47_CoCrFeNi, were found. Even though the actual eutectic compositions were very close to the predicted ones using the above simple methods, the former method was based on a known EHEA, which might limit its application [59] and for the latter, there should be a eutectic reaction between the added element and any element in the base alloy which is not always the case for EHEAs. For example, for the CoCrFeNiMnPd*_x_* EHEAs, eutectic reactions happen only in the Mn-Pd and Cr-Pd binary alloys while for the CoCrFeNiPdMn*_x_* EHEAs, eutectic reactions can be found only in the Pd-Mn binary alloy.

He et al. [42] designed a pseudo binary alloy, i.e., the CoCrFeNi HEA with a single FCC solid-solution phase as the base alloy and Nb as the additional element. Such simple pseudo binary method was followed by Jin et al. [59]. First, they chose one HEA with a single solid-solution phase and one stable binary intermetallic compound. After that, they obtained the HEA with dual phase by mixing the two phases. To ensure the formation of an eutectic structure, three conditions were proposed: (1) The single solid-solution phase should be stable enough without any segregation and precipitation; (2) the binary intermetallic compound should be stable from room temperature to its melting point; (3) the intermetallic compound must have the most negative mixing of enthalpy among all the binary combinations in the alloy. With CoCrFeNi_2_, Co_2_CrFeNi and CoCrFe_2_Ni as the HEAs with a single FCC solid-solution phase and NiAl as the binary intermetallic compound, they found three new EHEAs.

For the CoCrFeNiMnPd*_x_* and CoCrFeNiPdMn*_x_* EHEAs, CoCrFeNi can be taken to be the HEA with a single FCC solid-solution phase and MnPd can be taken to be the binary intermetallic compound; their mixing led to the CoCrFeNiMnPd EHEA [47]. Even though the mixing enthalpy between Mn and Pd was the most negative one (Table 4), the MnPd intermetallic compound was not stable enough from room temperature to its melting point [46]. As a result, the Mn*_x_*Pd*_y_* intermetallic compound in the eutectics depending on the compositions could be Mn_2_Pd_3_, Mn_3_Pd_5_ or Mn_7_Pd_9_ [46,47]. In one word, the pseudo binary method could be a simple way for designing EHEAs but the designing rules still need to be studied further to achieve general and effective rules. According to our study, the consistent elements in the EHEAs with a solid-solution phase and an intermetallic compound can be divided into two groups, i.e., two of them with very high mixing of enthalpy forms the intermetallic compound and the rest of them with very small mixing of enthalpy forms the solid-solution phase. There should be a eutectic reaction in the binary alloy system for the two elements in the first group. One of the eutectic phases is the solid-solution phase which should have a good solubility for all the elements in the second group. The other one is the intermetallic compound which might have negligible solubility for all the elements in the second group.

## 5. Conclusions

In the current work, the Mn*_x_* (*x* = 0, 0.2, 0.4, 0.6, 0.8) HEAs were prepared and characterized. Our main conclusions were as follows:

(1) With an increase in Mn addition, the microstructures of CoCrFeNiPdMn*_x_* HEAs changed from dendrites to divorced eutectics, to hypoeutectic microstructures and finally to eutectic dendrites. For the Mn_0.2_ and Mn_0.4_ (Mn_0.6_ and Mn_0.8_) EHEA, the FCC phase was a CoCrFeNiPd-rich (CoCrFeNi-rich) phase and the Mn*_x_*Pd*_y_* intermetallic compound was Mn_3_Pd_5_ (Mn_7_Pd_9_). Addition of Mn might influence the interface energy anisotropy of both the FCC/liquid and Mn*_x_*Pd*_y_*/liquid interfaces, thus forming the seaweed eutectic dendrites in the Mn_0.8_ EHEA.

(2) With an increase in Mn addition, the hardness of FCC phase increased first and then decreased. The solute strengthening effect of Mn and the formation of nanotwins made the hardness increase firstly, the suppression of nanotwins then decreased the hardness and finally the transition from the CoCrFeNiPd-rich to the CoCrFeNi-rich FCC phase made the hardness decrease considerably. For the Mn_3_Pd_5_ and Mn_7_Pd_9_ intermetallic compounds, a decrease of either *λ*_1_ or *λ*_2_ would increase the hardness.

(3) The current EHEA system violates to some extent all the designing rules for EHEAs. The pseudo binary method was improved accordingly and the current work might be helpful for accelerating designing of potential EHEAs.

## Figures and Tables

**Table 1:** EPMA results of the FCC phase in the CoCrFeNiPdMn*_x_* (*x* = 0–0.8) HEAs (in atomic fraction).

HEA	Co	Cr	Fe	Ni	Pd	Mn	FCC Phase
Mn_0.2_	22.32 ± 0.91	21.23 ± 0.48	19.30 ± 0.41	19.79 ± 0.28	13.49 ± 0.66	2.09 ± 0.36	CoCrFeNiPd-rich
Mn_0.4_	22.61 ± 0.53	21.92 ± 0.39	20.41 ± 0.90	20.02 ± 0.60	11.75 ± 0.67	3.56 ± 0.25	CoCrFeNiPd-rich
Mn_0.6_	22.29 ± 0.51	22.12 ± 0.76	21.32 ± 0.36	21.22 ± 0.97	8.09 ± 0.89	4.22 ± 0.45	CoCrFeNi-rich
Mn_0.8_	20.83 ± 0.36	21.44 ± 0.45	20.84 ± 0.53	24.46 ± 0.59	6.67 ± 0.70	5.12 ± 0.30	CoCrFeNi-rich

**Table 2:** EPMA results of the Mn*_x_*Pd*_y_* phase in the CoCrFeNiPdMn*_x_* (*x* = 0-0.8) HEAs (in atomic fraction).

HEA	Co	Cr	Fe	Ni	Pd	Mn	MnxPdy
Mn_0.2_	3.87 ± 0.85	7.48 ± 0.51	8.55 ± 0.17	5.49 ± 0.41	47.32 ± 0.65	27.29 ± 0.65	Mn_3_Pd_5_
Mn_0.4_	4.49 ± 0.83	7.32 ± 0.21	8.71 ± 0.21	5.53 ± 0.31	46.01 ± 0.76	29.44 ± 0.57	Mn_3_Pd_5_
Mn_0.6_	1.60 ± 0.09	4.78 ± 0.36	4.27 ± 0.45	3.39 ± 0.34	43.66 ± 0.93	42.31 ± 0.17	Mn_7_Pd_9_
Mn_0.8_	2.56 ± 0.38	6.28 ± 0.11	4.69 ± 0.79	3.81 ± 0.44	40.35 ± 0.67	41.71 ± 0.23	Mn_7_Pd_9_

**Table 3:** The measured spacing of primary twins (*λ*_1_) and secondary twins (*λ*_2_) for the Mn*_x_*Pd*_y_* phase in the CoCrFeNiPdMn*_x_* (*x* = 0.2–0.8) EHEAs.

EHEA (Mn_x_Pd_y_)	Spacing of Primary Twins *λ*_1_ (nm)	Spacing of Secondary Twins *λ*_2_ (nm)
Mn0.2 (Mn_3_Pd_5_)	242.10 ± 26.63	10.02 ± 1.10
Mn0.4 (Mn_3_Pd_5_)	180.33 ± 19.84	9.99 ± 1.22
Mn0.6 (Mn_7_Pd_9_)	14.96 ± 16.46	2.22 ± 0.24
Mn0.8 (Mn_7_Pd_9_)	15.02 ± 1.65	1.46 ± 0.16

**Table 4:** The mixing enthalpy $\Delta H_{mix}$ (kJ·mol^−1^) of atom pairs in the current CoCrFeNiPdMn*_x_* (*x* = 0.2–0.8) and some other EHEAs.

	Co	Cr	Ni	Mn	Pd	Al	Nb	Ta	Zr	Hf
Fe	−1	−1	−2	0	−4	−11	−16	−15	−25	−21
Co		−4	0	−5	−1	−19	−25	−24	−41	−35
Cr			−7	2	−15	−10	−9	−7	−12	−9
Ni				−8	0	−22	−30	−29	−49	−42
Mn					−23	−19	−4	−4	−15	−12
